# Urinary Dialkyl Phosphate Concentrations and Lung Function Parameters in Adolescents and Adults: Results from the Canadian Health Measures Survey

**DOI:** 10.1289/ehp.1509745

**Published:** 2015-09-15

**Authors:** Ming Ye, Jeremy Beach, Jonathan W. Martin, Ambikaipakan Senthilselvan

**Affiliations:** 1 School of Public Health, University of Alberta, Edmonton, Alberta, Canada; 2 Division of Preventive Medicine, Department of Medicine, University of Alberta, Edmonton, Alberta, Canada; 3 Division of Analytical and Environmental Toxicology, Department of Laboratory Medicine and Pathology, Faculty of Medicine & Dentistry, University of Alberta, Edmonton, Alberta, Canada

## Abstract

**Background:**

Epidemiological studies have reported associations between lung function parameters and organophosphate (OP) pesticide exposures in agricultural occupations, but to our knowledge associations have not been evaluated in general populations.

**Objectives:**

We examined associations between OP metabolite dialkyl phosphates (DAPs) and lung function using data from the Canadian Health Measures Survey (CHMS) Cycle 1.

**Methods:**

Forced vital capacity (FVC), forced expiratory volume in 1 sec (FEV1), FEV1/FVC ratio, and forced expiratory flow between 25% and 75% of FVC (FEF25%–75%) were measured for 4,446 CHMS participants. Urinary concentrations of six DAP metabolites (DMP, DMTP, DMDTP, DEP, DETP, and DEDTP), smoking status, and other predictors of lung function were also measured in the CHMS-Cycle 1. Multiple linear regression analyses were used to examine the relationship between total DAP concentrations (ΣDAPs) and lung function in adolescents (12–19 years) and adults (20–79 years).

**Results:**

In adults, estimates from multiple regression analyses suggested that a 1-unit increase on natural logarithmic scale (171% increase on the original scale) in the creatinine-corrected urinary concentration (nanomoles per gram creatinine) of ΣDAP was associated with a 32.6-mL (95% CI: –57.2, –8.1) reduction in FVC, 32.6-mL (95% CI: –59.0, –6.3) reduction in FEV1, 0.2% (95% CI: –0.6, 0.2) reduction in FEV1/FVC ratio, and 53.1-mL/sec (95% CI: –113.9, 7.7) reduction in FEF25%–75%. In adolescents, associations between ΣDAP and FEV1 were closer to the null and positive for FVC, whereas associations with FEV1/FVC and FEF25%–75% were negative, as in adults. However, none of the associations were significant in adolescents.

**Conclusions:**

The negative association between ΣDAP and lung function in adult participants suggests a detrimental effect of OP pesticides on lung function in the adult general population. Further studies using prospective designs are warranted to confirm the findings reported in this study.

**Citation:**

Ye M, Beach J, Martin JW, Senthilselvan A. 2016. Urinary dialkyl phosphate concentrations and lung function parameters in adolescents and adults: results from the Canadian Health Measures Survey. Environ Health Perspect 124:491–497; http://dx.doi.org/10.1289/ehp.1509745

## Introduction

Organophosphate (OP) pesticides have been used extensively in agricultural and residential applications. Humans may be exposed through occupational, environmental, and household exposure. Occupational exposures are mainly from agricultural occupations [U.S. Environmental Protection Agency (EPA) 2011], and environmental exposures can be from land run-off from the OP-treated areas (Kolpin et al. 1998). Mixing OP insecticides without personal protection can cause dermal absorption due to the high lipophilicity of OP inseticides (Reigart and Robert 1999). Ingestion of food and water contaminated with OPs is also a major route of exposure for general populations (Reigart and Robert 1999; Ye et al. 2015).

After entering the body, OP and/or its activated desulfurated “oxon” form (U.S. EPA 2006b), are rapidly hydrolyzed by phosphotriesterase paraoxonase 1 (PON1) to form dialkyl phosphate (DAP) metabolites that are subsequently excreted in the urine (Jansen et al. 2009). In the environment, generation of DAPs also occurs naturally when OP pesticides are degraded in soil, sediment, and surface water (Walker 2001).

There are six dialkyl phosphate metabolites: dimethyl phosphate (DMP), dimethyl thiophosphate (DMTP), dimethyl dithiophosphate (DMDTP), diethyl phosphate (DEP), diethyl thiophosphate (DETP), and diethyl dithiophosphate (DEDTP). These dialkyl phosphates are common metabolites of OPs, but are not pesticide specific (Kapka-Skrzypczak et al. 2011). Blood or urinary levels of DAPs are often considered as biomarkers of exposures to parental OP pesticides or their metabolites in the environment (Kapka-Skrzypczak et al. 2011). The detection of DAPs in urine samples is generally believed to reflect recent exposures to OPs over the previous few days (Kapka-Skrzypczak et al. 2011).

OP pesticides function as cholinesterase inhibitors and thus interfere with neural transmission in the nervous system (Androutsopoulos et al. 2013). In humans, health concerns related to OP exposures focus mainly on their high acute neurotoxicity (Keifer and Firestone 2007). Exposures to high doses of OPs can cause death due to respiratory paralysis and bradycardia (Keifer and Firestone 2007). Other adverse health effects associated with OP exposures include chronic neurological (Abou-Donia 2003), neurodevelopmental (Eskenazi et al. 2007), immunological (Corsini et al. 2008), endocrine-disruptive (De Coster and van Larebeke 2012), and respiratory effects (Ye et al. 2013). Because of these health concerns, OP pesticides have been largely restricted to use in agricultural applications in many countries. For example, chlorpyrifos has been banned for residential use in the United States since 2001 (U.S. EPA 2006a).

Exposures to OP insecticides in agricultural occupations have been associated with both respiratory symptoms and diseases. In a matched case–control study of 376 agricultural workers with 348 age- and sex-matched control subjects in Eastern India, exposures to OP insecticides were significantly associated with runny or stuffy nose, sore throat, dry cough, wheezing, breathlessness, chest tightness, and dyspnea (Chakraborty et al. 2009). The Agricultural Health Study conducted in the United States reported that exposures to OP insecticides chlorpyrifos and parathion were associated with wheezing and adult-onset asthma (Hoppin et al. 2002, 2006). In addition, the prevalence of chronic bronchitis has been associated with occupational exposures to OP insecticides among agricultural workers in India (Chakraborty et al. 2009) and in the United States (Hoppin et al. 2007; Valcin et al. 2007).

Few studies on OP exposures in agriculture have examined the association of OP pesticides on lung function. Peiris-John et al. reported that reductions in forced expiratory volume in 1 sec (FEV_1_) and forced vital capacity (FVC) were significantly associated with an OP insecticide application period among farm workers (*n* = 25, *p* < 0.05) in Sri Lanka (Peiris-John et al. 2005). In a study from India, exposures to OP insecticides among agricultural sprayers were significantly associated with lower mean FVC (sprayers vs. control: 2.23 L vs. 2.58 L), FEV_1_ (sprayers vs. control: 1.94 L vs. 2.36 L), FEV_1_/FVC ratio (sprayers vs. control: 87.0% vs. 91.5%), forced expiratory flow between 25% and 75% of FVC (FEF_25%–75%_) (sprayers vs. control: 2.25 L/sec vs. 2.81 L/sec) and peak expiratory flow rate (PEFR) (sprayers vs. control: 2.04 L/sec vs. 2.73 L/sec) among agricultural workers (*n* = 724, *p* < 0.0001) (Chakraborty et al. 2009). In addition, impaired lung function has been found to be associated with OP-induced cholinesterase inhibition (Fareed et al. 2013). A cross-sectional study of pesticide sprayers (*n* = 166) in India showed that impaired lung function was significantly correlated with lower activities of acetylcholinesterase (*p* < 0.01) and butylcholinesterase (*p* < 0.05), suggesting an adverse effect of OPs on lung function (Fareed et al. 2013).

Although there have been a few studies on the association between OP exposures and lung function in farm workers, to our knowledge, none have reported the impact of OP exposure on lung function in the general population. In the current study, urinary concentrations of dialkyl phosphate metabolites and their association with lung function were characterized among a Canadian general population.

## Methods

In this study, we used data from the first Canadian Health Measures Survey (CHMS-Cycle 1), a nationwide cross-sectional survey conducted by Statistics Canada in 2007–2009 (Statistics Canada 2011). Statistics Canada considered that the CHMS participants were a representative sample of the Canadian general population (Statistics Canada 2011). The study participants comprised 4,446 CHMS participants, including 980 adolescents 12–19 years of age and 3,466 adults 20–79 years of age, who had data available on urinary concentrations of DAPs (Health Canada 2010), spirometric measurements of lung function, smoking status, and other predictors of lung function.

The CHMS-Cycle 1 participants were chosen using a multi-stage sampling strategy. Collection sites were stratified by geographic region and by the census metropolitan area (CMA), and then selected according to the population size. Inhabitants of dwellings gave the household composition information of each dwelling. Dwellings in the collection sites were stratified by age groups according to the probability of having inhabitants with desired age in each dwelling, and then equal number of dwellings was sampled within each age stratum. Participants were then sampled from chosen dwellings in each age stratum (Statistics Canada 2011). People living on reserves and Aboriginal settlements, residents of institutions, members of the Canadian Forces, and those living in remote areas with population density that is too low were excluded (Statistics Canada 2011). The overall response rate was 51.7% for the CHMS-Cycle 1 (Statistics Canada 2011). A detailed description of the CHMS-Cycle 1 can be obtained from Statistics Canada (2011).

Participation in the CHMS was voluntary, and all 4,446 subjects provided written informed consent to store and use their urine samples (Statistics Canada 2011). This study was approved by the Health Research Ethics Board of the University of Alberta.

*Urinary concentrations of dialkyl phosphates.* Approximately 60 mL of mid-stream urine was collected from each CHMS participant using a urine specimen container. After collection, urine samples were refrigerated immediately and transported as soon as possible using refrigerated shipment to an analytical laboratory at the National Public Health Institute of Quebec (INSPQ) in Quebec City for analyzing DAP metabolites (Statistics Canada 2011). Concentrations of six DAP metabolites (DMP, DMTP, DMDTP, DEP, DETP, and DEDTP) were measured using gas chromatography–mass spectrometry (GC-MS) (Health Canada 2010; Statistics Canada 2011). Limits of detection (LOD) for measuring DMP, DMTP, DMDTP, DEP, DETP, and DEDTP were 7.9, 4.2, 1.9, 6.5, 3.5, and 1.6 nmol/L, respectively (Health Canada 2010). In addition, urinary creatinine concentration was measured using the colorimetric Jaffe method, and concentrations of DAPs were normalized to allow for urine dilution (Barr et al. 2005).

The total concentration of all six DAP metabolites (ΣDAP), the three dimethyl alkylphosphate metabolites (ΣDMAP), and the three diethyl alkylphosphate metabolites (ΣDEAP) was used to estimate the overall exposures to all OPs, OPs with DMAP metabolites such as malathion, and OPs with DEAP metabolites such as chlorpyrifos, respectively (Oulhote and Bouchard 2013). To calculate ΣDAP, ΣDMAP, and ΣDEAP, mass concentrations of DAPs in urine (micrograms per gram creatinine) were converted to molar concentrations (nanomoles per gram creatinine) using respective molecular weights of DAPs. Samples with DAP concentrations below the LOD were assigned as 0.5 × LOD (Hornung and Reed 1990).

*Lung function measurements.* Lung function parameters FVC, FEV_1_, FEV_1_/FVC ratio, and FEF_25%–75%_ were considered in the current study. Lung function tests were performed at the mobile examination center (MEC) on the same day when each participant’s urine sample was collected.

Trained technologists measured the lung function of participants using a portable flow-based spirometer (Koko®; PDS Instrumentation Inc., Louisville, CO, USA). American Thoracic Society (ATS) recommendations for performance of spirometry were followed, including calibrating spirometers with a 3-L syringe and obtaining a minimum of three acceptable trials from a maximum of eight maneuvers based on the ATS definition of within- and between-maneuver criteria for usable and acceptable trials (Miller et al. 2005; Statistics Canada 2011). Lung function measures were standardized to body temperature, barometric pressure, and water saturation (BTPS) measured at the MEC on the same day (Statistics Canada 2011).

For FVC and FEV_1_ measurements, the largest value of acceptable trials was used, and for FEF_25%–75%_ measurements, the mean flow rate (liters per second) of the acceptable trial with the largest sum of FVC and FEV_1_ was used (Statistics Canada 2011). Subjects with difficulty in breathing at rest, acute (e.g., cold, bronchitis, flu) or chronic respiratory condition (e.g., persistent cough), taking medication for tuberculosis, recent eye (within 6 weeks), chest or abdominal (within 3 months) surgery, pregnancy (> 27 weeks), or with an important language barrier were excluded from lung function testing (Statistics Canada 2011).

*Factors related to lung function.* Information on age, sex, and ethnicity were collected using the CHMS-Cycle 1 household questionnaire (Statistics Canada 2011). Standing height and weight were objectively measured by a fixed stadiometer with scales using a standard procedure based on the Canadian Physical Activity, Fitness and Lifestyle Approach (CPAFLA) (Statistics Canada 2011).

Information on tobacco smoking was obtained for participants > 12 years of age using the CHMS-Cycle 1 household questionnaire regarding the frequency (daily, occasionally, or not at all) and the duration of cigarette smoking (ages when subjects started smoking at least 1 cigarette/month and ages when they stopped smoking completely) (Statistics Canada 2011). Based on the responses to the questionnaire, a variable with three categories was defined by CHMS to indicate never, former smoker (including former daily and former occasional smoker), and current smoker, respectively. Pack-years, defined as number of packs of cigarettes smoked per day multiplied by number of years of smoking, were also calculated using detailed information collected on smoking in the CHMS-Cycle 1 (Statistics Canada 2011). In the pack-years calculation, never smokers and former occasional smokers (< 1 cigarette smoked/day in the past) were assigned a value of 0 pack-years.

Other lung function–related factors, including environmental tobacco exposure (exposed to secondhand smoke inside their home, in their private vehicle, in public places such as bars, restaurants, shopping malls, or at their place of work), types of heating source used at home (gas furnace/fireplace, oil furnace, electric heat, or wood-burning fireplace/stove), and air quality (ambient concentrations of particulate matter with diameter ≤ 2.5 μm (PM_2.5_), nitrogen dioxide (NO_2_), and ozone (O_3_) that were recorded hourly by the National Air Pollution Surveillance Program (http://www.ec.gc.ca/rnspa-naps/) on the same day when the spirometry tests were performed, were also measured as part of CHMS-Cycle 1 (Statistics Canada 2011).

*Statistical analyses.* Lung function parameters FVC, FEV_1_, FEV_1_/FVC, and FEF_25%–75%_ were considered as continuous outcome variables. Natural log–transformed total concentrations of dialkly phosphates (ΣDAPs, ΣDMAPs, and ΣDEAPs) were considered as continuous exposure variables in the analyses to reduce the skewness of the distribution, which leads to a 1-unit increase in log-transformed DAP concentrations equivalent to 171% increase in the actual concentrations.

In the descriptive analyses, geometric means, medians, interquartile ranges (IQRs) of urinary concentrations (nanomoles per gram creatinine), and proportions of subjects with detectable urinary concentrations (≥ LOD) of DAPs, were calculated for each DAP metabolite, as well as for ΣDAP, ΣDMAP, and ΣDEAP. Descriptive statistics were not calculated for individual metabolites that were below the LOD in > 40% of samples (Health Canada 2010). Demographic and anthropomorphic characteristics and smoking status of participants were described by means with standard errors (SE) or proportions.

We incorporated sampling design weights provided by Statistics Canada in our statistical analyses to adjust for poststratification in the multistage sampling, units with no responses, and out-of-scope responses (Statistics Canada 2011). To allow for the complex sampling design, 500 bootstrap weights, provided by Statistics Canada, were applied in variance estimation for descriptive statistics, regression coefficients, and 95% confidence intervals (CIs) (Statistics Canada 2011).

Bivariate analyses were initially conducted to examine the relationship between risk factors, including urinary concentrations of DAPs, and lung function. Factors that were significant at *p* ≤ 0.1 were considered in the multiple regression model for each individual lung function parameter. In multiple regression models, a purposeful selection method along with backward step-wise model building was used in determine the final models: That is, the variables that were consistently shown as predictors of lung function in the literature, including age, sex, ethnicity, height, and smoking, were forced into the final models. Other variables that were nonsignificant at *p* = 0.05 in the step-wise model building, including environmental tobacco exposure (yes/no), types of heating source (categorical variable) used at home, and ambient air pollutants (continuous concentrations of PM_2.5_, NO_2_, and O_3_) were excluded from the final models. Associations between urinary concentrations of DAPs (ΣDAP, ΣDMAP, or ΣDEAP) and lung function were determined by the final multiple linear regression models with lung function parameters as dependent variables and natural log–transformed creatinine-corrected urinary DAP concentrations as independent variables, adjusting for age (continuous), sex, ethnicity (Caucasian or other), height (continuous), smoking status (never, former, current), and weight (continuous). In addition, product interaction terms between urinary DAP concentrations and age (continuous), sex (male/female), ethnicity (Caucasian or other), and smoking status (never, former, current) on the association with lung function outcomes were also examined, with *p* ≤ 0.05 being considered in the final models. Separate regression models were used to examine the association among adolescent (12–19 years) and adult (20–79 years) participants, respectively.

Sensitivity analyses were performed using mass volume concentrations (nanomoles per liter) of ΣDAP as an exposure variable and adjusting for urinary creatinine concentration (grams per liter) as a separate independent covariate, instead of modeling creatinine-corrected ΣDAP (Barr et al. 2005).

Statistical analyses were performed using STATA (release 12; StataCorp, College Station, TX, USA) and SAS version 9.3 (SAS Institute Inc., Cary, NC, USA) software with procedures for the complex survey data analysis. In this study, we used default alpha level in STATA; that is, *p* ≤ 0.05 was considered as statistical significance.

## Results

*Characteristics of the study participants.* Demographic and anthropometric characteristics and smoking status of participants, are summarized in Table 1. Among 4,446 participants in the CHMS-Cycle 1 (2007–2009), 980 (22.0%) were adolescents (12–19 years) with a mean age of 15.5 years, and 3,466 (78.0%) were adults (20–79 years) with a mean age of 45.5 years. Overall, males and females were almost equally represented (Table 1). Approximately 62.7% of the participants ages 12–19 years and 71.7% of the participants ages 20–79 years self-identified as of Caucasian ethnicity. Although most adolescent participants (85.6%) had never smoked, approximately half of the adult participants had ever smoked at some time during their lifetime (Table 1).

**Table 1:** Characteristics of the study population by age group.*^a^*

Characteristic	Age groups (total *n *= 4,446)	
	12–19 years (*n *= 980)^*b*^	20–79 years (*n *= 3,466)^*b*^
Sex (%)
Female	48.8	50.5
Male	51.2	49.5
Height (cm)^*c*^	166.70 (0.24)	168.58 (0.28)
Weight (kg)^*c*^	63.16 (0.98)	77.65 (0.76)
Ethnicity (%)
Caucasian	62.7	71.7
Other	37.3	28.3
Province of residence (%)
New Brunswick	7.1	7.2
Quebec	23.3	23.7
Ontario	39.2	38.7
Alberta	16.9	16.8
British Columbia	13.5	13.6
Smoking status (%)
Never	85.6	47.9
Former	2.4	30.6
Current	12.0	21.5
Pack-years for smokers^*c*^	1.00 (0.20)	9.90 (0.42)
^***a***^Survey design weights and 500 bootstrap weights were used in calculating percentages, mean values, and standard errors. ^***b***^Among 4,446 participants, 22.0% were adolescents 12–19 years, and 78.0% were adults 20–79 years of age. ^***c***^Mean (SE).		

*Lung function among the study participants.* Lung function parameters across demographic groups and smoking status are summarized in Table 2. In adults, lung function was larger in young adults (age 20–29 years) than in older adults (30–79 years) (Table 2). Although there was no significant difference in the mean lung function measures between smoking categories in the univariate analyses, after adjusting for age, sex, ethnicity, weight, and height, for both adolescent and adult participants, former and current smokers had statistically lower mean values of FEV_1_, FEV_1_/FVC ratio, and FEF_25%–75%_ than did nonsmokers (*p* < 0.01, data not shown). After controlling for age, sex, ethnicity, weight, height, and smoking status, lung function parameters followed a reasonably normal distribution for the CHMS participants (data not shown). In addition, there were no significant associations in adolescents or adults between lung function parameters and exposure to environmental tobacco smoking, types of heating source used at home, or ambient concentration of PM_2.5_, NO_2_, and O_3_ (data not shown).

**Table 2:** Distribution of the means of lung function parameters by demographic factors and smoking status by age group (mean ± SE).

Characteristic	FVC (L)	FEV_1_ (L)	FEV_1_/FVC (%)	FEF_25%–75%_ (L/sec)
12–19 years (*n *= 980)
Total sample	4.14 ± 0.04	3.46 ± 0.03	84.0 ± 0.3	3.55 ± 0.05
Sex
Female (reference)	3.71 ± 0.03	3.16 ± 0.03	85.5 ± 0.3	3.39 ± 0.06
Male	4.54 ± 0.07*	3.74 ± 0.05*	82.6 ± 0.5*	3.69 ± 0.07*
Ethnicity
Caucasian (reference)	4.28 ± 0.03	3.55 ± 0.02	83.4 ± 0.3	3.60 ± 0.06
Others	3.88 ± 0.07*	3.29 ± 0.05*	85.2 ± 0.5*	3.45 ± 0.06*
Smoking status
Never (reference)	4.06 ± 0.03	3.41 ± 0.03	84.3 ± 0.3	3.52 ± 0.05
Former smoker	4.36 ± 0.13*	3.58 ± 0.07*	82.5 ± 2.0*	3.65 ± 0.17*
Current smoker	4.61 ± 0.10*	3.77 ± 0.09*	82.3 ± 1.0*	3.71 ± 0.20*
20–79 years (*n *= 3,466)
Total sample	4.13 ± 0.04	3.19 ± 0.03	77.1 ± 0.3	2.88 ± 0.04
Age group
20–29 years (reference)	4.71 ± 0.10	3.81 ± 0.08	81.4 ± 0.5	3.69 ± 0.12
30–79 years	4.00 ± 0.03*	3.05 ± 0.02*	76.2 ± 0.3*	2.70 ± 0.03*
Sex
Female (reference)	3.45 ± 0.02	2.68 ± 0.02	77.6 ± 0.3	2.48 ± 0.04
Male	4.81 ± 0.05*	3.69 ± 0.04*	76.6 ± 0.4*	3.28 ± 0.06*
Ethnicity
Caucasian (reference)	4.19 ± 0.03	3.20 ± 0.03	76.3 ± 0.3	2.84 ± 0.04
Others	3.98 ± 0.06*	3.14 ± 0.04*	79.1 ± 0.5*	2.97 ± 0.04*
Smoking status
Never (reference)	4.12 ± 0.07	3.26 ± 0.05	79.2 ± 0.3	3.09 ± 0.06
Former smoker	4.02 ± 0.05*	3.06 ± 0.04*	75.9 ± 0.3*	2.67 ± 0.06*
Current smoker	4.30 ± 0.05*	3.21 ± 0.04*	74.4 ± 0.4*	2.72 ± 0.05*
Survey design weights and 500 bootstrap weights were used in calculating arithmetic mean values and standard errors. *Statistically significant differences in lung function from the reference group with *p *< 0.01.				

*Urinary concentrations of dialkyl phosphates in the study participants.* Among the total study participants, 91.3% had at least one of the six dialkyl phosphate metabolites detectable in their urine samples. The mean (both arithmetic and geometric means) concentrations (creatinine-adjusted) of DAP metabolites were higher among adults than adolescents (Table 3).

**Table 3:** Distribution of creatinine-corrected urinary concentrations of organophosphate metabolites in the study population by age group.

Organophosphate pesticide metabolites	Detection limit (nmol/L)	Percentage ≥ LOD^*a*^ (%)	AM (SE)^*b*^ (nmol/g)	GM (SE)^*b*^ (nmol/g)	Median (SE)(nmol/g)	IQR(nmol/g)
12–19 years (*n *= 980)
DMP	7.9	82.3	51.5 (3.0)	27.1 (2.1)	29.0 (2.7)	13.2–63.0
DMTP	4.2	68.6	55.0 (6.3)	14.0 (1.3)	14.1 (1.5)	< LOD–42.9
DMDT	1.9	35.4	—	—	< LOD	< LOD–3.6
DEP	6.5	82.0	28.1 (2.0)	16.8 (1.3)	16.9 (1.3)	9.0–33.7
DETP	3.5	44.6	—	—	< LOD	< LOD–< LOD
DEDT	1.6	4.4	—	—	< LOD	< LOD–< LOD
ΣDAP	NA	93.8	151.8 (12.0)	83.8 (5.5)	78.2 (7.8)	39.9–167.5
ΣDMAP	NA	88.4	116.0 (9.9)	51.6 (3.6)	48.4 (4.6)	22.3–119.5
ΣDEAP	NA	83.1	35.7 (2.5)	22.6 (1.5)	21.8 (1.8)	12.4–41.9
20–79 years (*n *= 3,466)
DMP	7.9	76.4	53.3 (3.5)	27.5 (2.0)	27.5 (2.0)	13.1–56.9
DMTP	4.2	66.7	68.8 (5.0)	17.2 (1.3)	14.5 (1.6)	< LOD–55.4
DMDT	1.9	36.4	—	—	< LOD	< LOD–5.5
DEP	6.5	77.9	27.6 (1.0)	17.5 (0.8)	17.9 (1.0)	9.8–33.4
DETP	3.5	36.1	—	—	< LOD	< LOD–< LOD
DEDT	1.6	2.3	—	—	< LOD	< LOD–< LOD
ΣDAP	NA	91.0	169.7 (9.5)	90.5 (4.5)	84.8 (5.1)	43.2–175.1
ΣDMAP	NA	82.4	132.7 (8.9)	55.5 (3.7)	50.7 (3.7)	23.2–125.0
ΣDEAP	NA	78.6	36.9 (1.1)	24.3 (0.9)	24.3 (1.0)	13.6–43.4
Abbreviations: AM, arithmetic means. GM, geometric means; NA, not applicable. Survey design weights and 500 bootstrap weights were used in calculating percentages, mean values, standard errors, and 95% confidence intervals.^***a***^Percentage of participants with at least one of the organophosphate metabolites in the sum ≥ LOD. ^***b***^If < 60% of samples had detectable organophosphate metabolites, means were not calculated.						

DMP and DEP were the most prevalent DAP metabolites (detected in approximately 80.0% of both adolescent and adult participants), whereas DEDTP was the least prevalent metabolite (detected in < 5.0% of adolescent and adult participants) (Table 3). In addition, the mean concentrations (creatinine-adjusted) of ΣDMAP were significantly higher than the ΣDEAP in both adolescent and adult participants (Table 3).

The geometric mean concentrations of ΣDAP were 83.8 ± 5.5 nmol/g creatinine and 90.5 ± 4.5 nmol/g creatinine for adolescent participants and adult participants, respectively (Table 3). In both adolescent and adult participants, females had significantly higher mean concentrations of DAP metabolites, including ΣDAP, ΣDMAP, and ΣDEAP, than male participants (*p* < 0.05) (Table 4). In addition, among adult participants, current smokers had statistically significant lower mean concentrations of DAP metabolites (ΣDAP, ΣDMAP, and ΣDEAP) than former smokers and the participants who never smoked (for current vs. never and current vs. former smokers, *p*-values < 0.01; Table 4). In both adolescents and adults, no significant difference in the mean concentrations of DAP metabolites was observed between Caucasians and participants in other ethnic groups (Table 4).

**Table 4:** Distribution of creatinine-corrected urinary concentrations (nmol/g creatinine) of organophosphate metabolites by demographic factors and smoking status by age group [GM (SE)].

Characteristic	12–19 years (*n *= 980)			20–79 years (*n *= 3,466)		
	ΣDAP	ΣDMAP	ΣDEAP	ΣDAP	ΣDMAP	ΣDEAP
Average	83.8 (5.5)	51.6 (3.6)	22.6 (1.5)	90.5 (4.5)	55.5 (3.7)	24.3 (0.9)
Sex
Female (reference)	95.3 (7.4)	59.9 (5.0)	24.3 (2.1)	112.2 (6.6)	68.5 (5.8)	29.8 (1.3)
Male	74.4 (5.3)*	44.9 (3.7)*	21.2 (1.2)*	72.6 (3.7)*	44.7 (2.9)*	19.7 (0.7)*
Ethnicity
Caucasian (reference)	84.5 (7.3)	51.2 (4.7)	23.5 (2.0)	90.4 (4.4)	55.5 (3.6)	24.7 (1.0)
Others	83.6 (5.9)	53.2 (4.1)	21.2 (1.4)	91.4 (6.8)	56.1 (5.4)	23.4 (1.5)
Smoking status
Never (reference)	87.8 (6.3)	54.3 (4.2)	23.4 (1.7)	96.7 (6.1)	60.2 (4.7)	25.3 (1.3)
Former smoker	70.8 (33.7)	46.7 (27.6)	16.7 (3.3)	104.0 (7.5)	64.5 (5.5)	26.0 (1.5)
Current smoker	61.8 (9.5)	36.3 (9.3)	18.7 (2.4)	63.8 (4.8)	37.3 (3.4)	20.2 (1.1)
GM, geometric mean. Survey design weights and 500 bootstrap weights were used in calculating geometric mean values and standard errors. *Statistically significant differences in DAP concentrations from the reference group with *p *< 0.05.						

*Relationships between DAP concentrations and lung function.* In the multiple regression analyses of adult participants 20–79 years of age, a 1-unit increase on natural logarithmic scale (171% increase on the original scale) in the creatinine-corrected urinary concentration of ΣDAP was associated with a 32.63-mL (95% CI: –57.21, –8.05) reduction in FVC, a 32.66-mL (95% CI: –59.02, –6.28) reduction in FEV_1_, 0.18% (95% CI: –0.61%, 0.24%) reduction in FEV_1_/FVC ratio, and 53.11-mL/sec (95% CI: –113.90, 7.68) reduction in FEF_25%–75%_ after adjusting for age, sex, ethnicity, height, weight, and smoking status (Table 5). In addition, urinary concentrations of ΣDMAP and ΣDEAP were also negatively associated with FVC and FEV_1_ in adults, but associations were statistically significant for ΣDMAP only (Table 5). No interactions between DAP concentrations and age, sex, ethnicity, or smoking status were significant at *p* ≤ 0.05 (data not shown). In addition, model estimates were similar when adjusting for pack-years as an untransformed continuous variable instead of smoking status (data not shown).

**Table 5:** Association between natural log–transformed creatinine-corrected urinary concentrations (nmol/g creatinine) of ΣDAP, ΣDMAP, and ΣDEAP and lung function parameters by age group.

Concentration	FVC (mL)		FEV_1_ (mL)		FEV_1_/FVC (%)		FEF_25–75%_ (mL/sec)	
	β (95% CI)	*p*-Value	β (95% CI)	*p*-Value	β (95% CI)	*p*-Value	β (95% CI)	*p*-Value
12–19 years (*n *= 980)
ΣDAP	13.93 (–24.37, 52.24)	0.44	–2.36 (–35.74, 31.02)	0.88	–0.33 (–0.86, 0.20)	0.20	–27.05 (–114.95, 60.85)	0.51
ΣDMAP	12.34 (–18.19, 42.87)	0.39	1.12 (–23.00, 25.24)	0.92	–0.22 (–0.68, 0.24)	0.31	–17.88 (–87.58, 51.82)	0.58
ΣDEAP	21.58 (–46.07, 89.22)	0.50	2.75 (–49.32, 54.83)	0.91	–0.34 (–0.86, 0.18)	0.18	–6.26 (–93.57, 81.05)	0.88
20–79 years (*n *= 3,466)
ΣDAP	–32.63 (–57.21, –8.05)	0.014	–32.65 (–59.02, –6.28)	0.02	–0.18 (–0.61, 0.24)	0.36	–53.11 (–113.90, 7.68)	0.081
ΣDMAP	–24.29 (–45.39, –3.18)	0.028	–24.18 (–45.52, –2.85)	0.03	–0.15 (–0.51, 0.21)	0.39	–38.73 (–90.26, 12.80)	0.13
ΣDEAP	–20.38 (–57.16, 16.39)	0.25	–26.34 (–61.08, 8.39)	0.12	–0.20 (–0.55, 0.14)	0.22	–62.17 (–113.57, –10.76)	0.022
Associations were characterized by the multiple linear regression analyses with controlling for age, sex, ethnicity, height, weight, and smoking status. Survey design weights and 500 bootstrap weights were included in calculating β coefficients and 95% CIs.								

In adolescents, associations between urinary DAP concentrations (including ΣDAP, ΣDMAP, and ΣDEAP) and lung function parameters were positive for FVC, close to null for FEV_1_, and negative for FEV_1_/FVC and FEF_25%–75%_ after adjusting for age, sex, ethnicity, height, weight, and smoking status. However, none of the associations were statistically significant in adolescents (Table 5).

Associations based on models of ΣDAP (nanomoles per liter urine), as well as ΣDMAP (nanomoles per liter urine) and ΣDEAP (nanomoles per liter urine) that included creatinine as a covariate were consistent with findings from models of creatinine-adjusted DAP concentrations (nanomoles per gram creatinine) for adolescent and adult participants (data not shown).

## Discussion

Our results also showed that > 90% of the CHMS-Cycle 1 participants 12–79 years of age had at least one species of DAP metabolite detectable in their urine. The prevalence and geometric mean concentration of most of the DAP metabolites reported in the present study (CHMS 2007–2009) were higher than those reported in a similar year for the U.S. population [National Health and Nutrition Examination Survey (NHANES) 2007–2008] [Centers for Disease Control and Prevention (CDC) 2015]. For example, the geometric means of urinary concentrations of DMP and DEP were 51.5 nmol/g and 28.1 nmol/g for participants of this study, but below the LOD in the U.S. population (NHANES 2007–2008) (CDC 2015), which may have resulted from a higher proportion of OP pesticide exposures in Canada than in the United States when assuming high correlation between the biomonitoring concentrations and exposures to OP pesticides. Given the short half-life of most OP pesticides in the environment (Pehkonen and Zhang 2002) and the short elimination half-life in humans (Kapka-Skrzypczak et al. 2011), the detection of DAP metabolites among most participants suggests that exposures to OP pesticides are common and ongoing in the Canadian general population.

In the present study, we estimated associations between urinary concentration of DAPs and lung function parameters among CHMS-Cycle 1 participants 12–79 years of age, a representative sample of the Canadian adolescents and adults (Statistics Canada 2011). To the best of our knowledge, the current study is the first nationwide population-based investigation on the relationships between DAP metabolites and lung function among the Canadian general population.

Among the adult participants, urinary concentrations of ΣDAP were significantly associated with the reduction in FVC and FEV_1_. The differences in FVC and FEV_1_ between adult participants at the 25th (43.2 nmol/g) and 75th (175.1 nmol/g) percentiles of urinary concentrations of ΣDAP, calculated by the product of beta coefficient for FVC or FEV_1_ in Table 5 with log (25th percentile of concentration/75th percentile of concentration), would be 45.67 mL and 45.70 mL, respectively, which is around 1–2% of typical lung function. The ΣDAP-associated 45.67-mL and 45.70-mL reduction in FVC and FEV_1_, respectively (after adjusting for age, sex, ethnicity, height, weight, and smoking status in this cross-sectional study) was similar in size to the natural age-related decline of lung function per year for healthy nonsmoking adults (approximately 30 mL/year in FVC and 20–30 mL/year in FEV_1_) (Burrows et al. 1983; Peat et al. 1990). Nevertheless, the magnitude of any DAP concentration–associated lung function reduction would be better characterized in studies with longitudinal designs.

Results from the multiple regression analyses showed significant association between DAP concentrations and lung function in adult participants (20–79 years) but not in adolescent participants (12–19 years). The reasons for this difference are unclear; but perhaps the rapid growth of the lungs during the growth spurt in adolescents increases the variance in lung function parameters (Pellegrino et al. 2005), which will result in larger uncertainties in characterizing the association between insecticide exposures and lung function. This difference could also be attributable to differences in the magnitude, including duration and/or timing, of OP exposures, uncontrolled confounding effect, the smaller sample size of adolescent versus adult participants, and/or any other sources of bias that lead to differences between adults and adolescents.

Although both methyl DAPs and ethyl DAPs were negatively associated with lung function among adults, they were only statistically significant for ΣDMAP. This result could be attributable to less variation (smaller IQR) in ΣDEAP concentrations. In addition, a higher mean concentration of ΣDMAP than ΣDEAP might suggest that the potential OP-related lung function changes identified could stem mainly from the OP insecticides with methyl DAP metabolites, although future work is required to confirm this.

Several studies in the literature have suggested that exposures to OP pesticides in agricultural occupations were associated with a reduction in lung function parameters, including FEV_1_ (Chakraborty et al. 2009; Fareed et al. 2013; Peiris-John et al. 2005), FVC (Chakraborty et al. 2009; Peiris-John et al. 2005), FEV_1_/FVC ratio (Chakraborty et al. 2009; Fareed et al. 2013), FEF_25%–75%_ (Chakraborty et al. 2009), and peak expiratory flow rate (Chakraborty et al. 2009; Fareed et al. 2013). In our study, the urinary ΣDAP level was associated with reductions in both FVC and FEV_1_ among the adult general population, which is consistent with the findings in agricultural occupations (Chakraborty et al. 2009; Fareed et al. 2013; Peiris-John et al. 2005).

OP pesticides are neurotoxicants that bind to the serine residue in acetylcholine esterase (AChE), resulting in an accumulation of acetylcholine (ACh) and overstimulation of postsynaptic cholinergic nerves (Androutsopoulos et al. 2013).This suggests a number of possible mechanisms by which OP exposure could affect lung function. Muscarinic 3 (M3) receptors, a stimulatory type of muscarinic ACh receptors, are expressed on both pulmonary nerves and smooth muscles (Racké and Matthiesen 2004). Stimulation of M3 receptors by ACh would potentially lead to the contraction of airway smooth muscles *ex vivo* (Fryer and Jacoby 1998). Another muscarinic receptor—the M2 receptor, located on the pulmonary prejunctional nerves and smooth muscles—can inhibit further release of ACh from prejunctional nerve ends (Costello et al. 1998). Based on studies of guinea pigs, at a low-dose level (which may be particularly relevant among general populations), OP pesticides do not seem to inhibit AChE but have the potential to disrupt the auto-inhibitory function of pulmonary prejunctional M2 receptors (Lein and Fryer 2005; Proskocil et al. 2010). This can lead to an unopposed release of ACh from prejunctional parasympathetic nerves, which again might cause excessive bronchoconstriction (Minette and Barnes 1988). Nevertheless, mechanisms of OP-associated lung function reduction in humans may not be the same as these experimental findings.

Inhalation of OP-containing gases, vapors, or aerosols into airways can also lead to production of reactive oxygen species (ROS) and subsequent activation of a series of stress-responsive signalling pathways, including ERK (extracellular-signal-regulated kinase)–MAPK (mitogen-activated protein kinase), JNK (c-Jun N-terminal kinases), and NF-κB (nuclear factor kappa-light-chain-enhancer of activated B cells) signalling pathways (Terry 2012). This may in turn cause contraction of airway smooth muscles and airway narrowing (Abdollahi et al. 2004; Tomasic et al. 1992).

Notwithstanding our consideration of these actions of OPs, the biological mechanisms underlying any OP-related reduction of lung function remain unclear. The proposed biological mechanisms are not exclusive for any specific type of pulmonary diseases. Further study to characterize the biological plausibility of the OP-associated type of lung function impairment is necessary.

For the general population, dietary intake of trace amounts of OPs from pesticide-sprayed or -treated fruits and vegetables is a major source of OP exposures (Reigart and Robert 1999). However, as suggested by Zhang et al. (2008), ingestion of environmentally preformed DAPs can also lead to the detection of DAP metabolites in urine samples. Therefore, in addition to OP pesticides, DAP metabolites detected in urine samples in the current study may have resulted from the direct exposure to environmental DAPs, which are currently not known to be toxic to human health.

There are several limitations in our data. Firstly, data from the CHMS did not cover the entire Canadian population. Aboriginal people living on reserves and Aboriginal settlements, people living in remote areas, institutional residents, and full-time members of the Canadian Armed Forces were excluded from the CHMS-Cycle 1 (Statistics Canada 2011). However, it is unlikely that the exclusion of these groups would change the relationships reported in this study, because the excluded populations in the CHMS-Cycle 1 represent < 4% of the total Canadian population (Statistics Canada 2011). Second, subjects with chronic or acute respiratory conditions and taking medication for tuberculosis (Statistics Canada 2011) were excluded from the regression analyses, which will limit our ability to generalize our results to a broader population. Third, although urinary levels of DAP metabolites can be considered as an objective measure of actual body burden arising from OP pesticide exposures (Kapka-Skrzypczak et al. 2011), they lack specificity in identifying corresponding pesticides; therefore, the present study was not able to provide information on specific OP pesticides that the participants were exposed to. Moreover, this study cannot distinguish between environmentally preformed DAPs and metabolite DAPs resulting from exposure to the parent compounds. Last, because of the cross-sectional nature of the CHMS, our data provide only a snapshot of urinary DAP concentrations; repeated DAP measurements were not conducted longitudinally during the year, which may not be directly related to peak or cumulative OP exposures (Kapka-Skrzypczak et al. 2011), and it was also not possible to examine the potential effect of measurement variability on the results. Moreover, the temporal sequence between changes in lung function and exposures to OPs cannot be determined in the present study.

## Conclusions

Our results showed that urinary concentrations of total DAPs were significantly associated with reductions in FVC and FEV_1_ among adult participants 20–79 years of age, a representative sample of the Canadian general population.

Although many OP pesticides have been restricted for agricultural uses only, exposures remain common and may still pose risks to public health (U.S. EPA 2006a). Further research using prospective designs is warranted to confirm the associations reported in this study.
